# *NLRP3* Inflammasome Polymorphism and Macrovascular Complications in Type 2 Diabetes Patients

**DOI:** 10.1155/2015/616747

**Published:** 2015-07-27

**Authors:** Jasna Klen, Katja Goričar, Andrej Janež, Vita Dolžan

**Affiliations:** ^1^General Hospital Trbovlje, Rudarska cesta 9, SI-1420 Trbovlje, Slovenia; ^2^Pharmacogenetics Laboratory, Institute of Biochemistry, Faculty of Medicine, University of Ljubljana, Vrazov trg 2, SI-1000 Ljubljana, Slovenia; ^3^Department of Endocrinology, Diabetes and Metabolic Diseases, University Medical Center Ljubljana, Zaloška Cesta 7, SI-1000 Ljubljana, Slovenia

## Abstract

*Background*. It is generally accepted that poor glycemic control, arterial hypertension and/or hyperlipidemia, and the associated oxidative stress may contribute to the development of macro- and microvascular complications in type 2 diabetes (T2D). Such metabolic damage signals may activate inflammasome and trigger chronic inflammation. We investigated common polymorphisms in inflammasome coding genes and the risk for macro- and microvascular complications in T2D. *Methods*. In total 181 clinically well-characterised T2D patients were genotyped for *NLRP3* rs35829419 and *CARD8* rs2043211. Risk for diabetic complications was assessed using logistic regression. *Results*. Patients with median duration of T2D 11 (6–17) years had relatively well controlled blood glucose and lipid levels and blood pressure on the prescribed treatment regimen. Duration of T2D and plasma cholesterol levels were the most important clinical risk factors for macrovascular complications (*P* = 0.007 and *P* = 0.031). *NLRP3* rs35829419 was associated with increased risk for macrovascular complications (*P* = 0.004), with myocardial infarction in particular (*P* = 0.052). No association was observed between *CARD8* polymorphism and any of T2D complications. *Conclusions*. Our preliminary data suggest the role of *NLRP3* polymorphism in diabetic macrovascular complications, especially in myocardial infarction.

## 1. Introduction

Macro- and microvascular complications are common in long-standing type 2 diabetes (T2D) and diminish the quality of life and life-time expectancy of patients [1, 2]. In addition to genetic and lifestyle factors, poor glycemic control, arterial hypertension, and/or hyperlipidemia are predisposing factors for the development of these complications [3, 4]. High blood glucose levels and dyslipidemia may stimulate the production of reactive oxygen species (ROS), leading to increased oxidative stress and chronic inflammation that appears to be the major driver of molecular processes leading to late diabetic complications [5–8]. Recent studies suggested the involvement of inflammatory pathways that may be triggered by inflammasome, a crucial complex that senses and is activated by metabolic damage signals [9, 10].

Inflammasomes are a large family of multiprotein complexes that can be activated by pathogen-associated or damage-associated molecular patterns (PAMPs or DAMPs, resp.), among others also such as ROS, cholesterol crystals, and possibly high glucose levels. NLRP3 inflammasome is composed of the NLRP3 scaffold protein, CARD containing adaptor protein, and caspase-1 [11]. Activation of inflammasome leads to assembly of the multiprotein complex that cleaves and activates caspase-1, resulting in cleavage of pro-interleukin-1*β* (pro-IL-1*β*) and release of IL-1*β* that triggers downstream inflammatory response [9, 10].

NLRP3 inflammasome has been suggested as a link between insulin resistance, obesity, circulating immune markers, immunogenetic susceptibility, macrophage function, and chronic inflammation [12]. Genetic variations leading to the altered production or function of inflammasome and inflammatory cytokines were linked to various inflammatory diseases, including obesity, insulin resistance, and T2D [13, 14]. Two inflammasome related single nucleotide polymorphisms (SNPs) have been most frequently studied in Caucasians.* NLRP3* rs35829419 (p.Gln705Lys) is a gain-of-function polymorphism associated with increased production of IL-1*β* [15]. On the other hand,* CARD8 *rs2043211 (p.Cys10Ter) results in nonfunctional protein and leads to loss of CARD-8 inhibition of caspase-1. Both SNPs were associated with proinflammatory phenotype [16–19]. Another common SNP associated with mRNA stability,* NLRP3* rs10754558, was linked to insulin resistance and increased risk for T2D in Chinese Han population [13].

The aim of our study was to investigate if* NLRP3* and* CARD8* polymorphisms play a role in the risk for macro- and microvascular complications in T2D.

## 2. Patients and Methods

We performed a retrospective study that included T2D patients aged between 18 and 75 years. All the patients were treated and followed up at the outpatient clinic at the General Hospital Trbovlje, Slovenia. Patients with diabetes type 1, gestational diabetes, other types of diabetes, active cancer, addiction to alcohol or illegal drugs, and dementia or severe psychiatric disorders were not eligible for the study. Patients were included as they were coming for their regular follow-up visits and were receiving treatment according to the established clinical guidelines. At inclusion they all underwent a complete physical examination and laboratory evaluation. Blood pressure, body height, and body weight were measured at every follow-up visit and the body mass index (BMI) was calculated. Information on the history of diabetes, presence of arterial hypertension, hyperlipidemia and chronic macrovascular and microvascular diabetic complications, smoking status, and other medications was obtained from the medical records and from the interview at the inclusion in the study. Plasma lipid levels, urea and creatinine, and urine albumin and albumin/creatinine ratio were determined at least once per year. All patients were referred to consulting ophthalmologist for screening for diabetic retinopathy at least once a year. Echosonography and exercise stress test (cycloergometry) were performed at the first visit and also at any complaints suggestive for ischemic heart disease.

The study was approved by the National Ethics Committee and conducted in accordance with the Declaration of Helsinki. Written informed consent was obtained from all subjects.

All the laboratory parameters were measured using standard laboratory procedures at the biochemistry laboratory of the General Hospital Trbovlje, Slovenia [20]. Genomic DNA was isolated from peripheral blood leukocytes using Qiagen FlexiGene kit (Qiagen, Hilden, Germany). DNA extraction and genotyping analyses were performed at the Pharmacogenetics Laboratory, Institute of Biochemistry, Faculty of Medicine, University of Ljubljana. Genotyping of* NLRP3* rs35829419 (c.2113C>A, p.Gln705Lys) and* CARD8 *rs2043211 (c.304A>T, p.Phe102Ile, p.Cys10Ter) was carried out using a fluorescence-based competitive allele-specific (KASPar) assay according to the manufacturer's instructions (KBiosciences, Herts, UK). Genotyping was performed blindly to any clinical data and was randomly repeated in 20% of samples to check for genotyping reliability.

Median and interquartile range were used to describe central tendency and variability of continuous variables, while frequencies were used to describe the distribution of categorical variables. Standard chi square test or Mann-Whitney test was used to compare clinical characteristics between different patient groups. Risk for macro- and microvascular complications was assessed using logistic regression. All statistical analyses were performed using IBM SPSS Statistics 19.0 (IBM Corporation, Armonk, NY, USA). Dominant genetic model was used in all analyses and the level of statistical significance was set at 0.05.

## 3. Results

In total 181 patients (105 males and 76 females) with T2D were included in the study. The median (25–75% range) age of the patients was 64 (58.5–70.5) years and duration of T2D was 11 (6–17) years. On the prescribed treatment regimen most of the patients achieved relatively good blood glucose control with the average HbA1c 6.9% (6.3–7.6), well controlled blood pressure, and plasma lipid levels (Table 1).

In total 46 (25.4%) patients suffered from macrovascular complications: 10 (5.5%) had peripheral arterial occlusive disease (PAOD), 16 (8.85%) ischemic cerebrovascular disease (ICD), and 32 (17.7%) myocardial infarction (MI). Only 34 (18.8%) patients had microvascular complications: 25 (13.8%) suffered from end-stage kidney failure due to diabetic nephropathy, 13 (7.2%) from neuropathy, and 15 (8.3%) from retinopathy. Duration of T2D and plasma cholesterol levels were the most important clinical risk factors for macrovascular complications in logistic regression analysis (OR = 1.06; 95% CI = 1.02–1.11; *P* = 0.007 and OR = 0.67; 95% CI = 0.46–0.96; *P* = 0.031, resp.). The most important clinical risk factor for microvascular complications was duration of T2D (OR = 1.12; 95% CI = 1.07–1.17; *P* < 0.001).

All the patients were genotyped for* CARD8 *rs2043211 and* NLRP3* rs35829419 polymorphisms; however, CARD8 genotype data could not be obtained for one patient. In total 89 (49.4%) patients were homozygous for the wild type* CARD8* AA allele, 70 (38.9%) carried one, and 21 (11.7%) carried two polymorphic T alleles.* NLRP3* polymorphism was less common: 160 (88.4%) were homozygous for the wild type CC genotype, while 21 (11.6%) patients carried one of the polymorphic A alleles: 20 (11.0%) carried one and 1 (0.6%) carried two A alleles. All genotype frequencies were in agreement with HWE (*P* = 0.213 and 0.666 for* CARD8 *and* NLRP3, *resp.).

We observed an association between* NLRP3* rs35829419 polymorphism and the risk for macrovascular complications (OR = 3.93; 95% CI = 1.54–10.0; *P* = 0.004) (Table 2). This association remained significant after adjustment for duration of T2D and plasma cholesterol levels (OR = 4.36; 95% CI = 1.58–12.01; *P* = 0.004). Although carriers of one or two polymorphic* NLRP3* rs35829419 alleles had higher risk for MI, PAOD, and ICD, these associations were statistically nonsignificant (*P* = 0.052, *P* = 0.078, and *P* = 0.092, resp., Table 2). When stratified by sex, a significant association of* NLRP3* rs35829419 with macrovascular complications was observed only in males (OR = 4.44; 95% CI = 1.39–14.25; *P* = 0.012) (Table 2). Additionally, significant association with PAOD was observed among males (OR = 5.93; 95% CI = 1.17–30.06; *P* = 0.032). This association could not be tested in females as only 3 female patients had PAOD and all were* NLRP3* CC homozygous.

No association was observed between the investigated* CARD8* rs2043211 and the risk for macrovascular complications in univariable (OR = 0.97; 95% CI = 0.50–1.90; *P* = 0.930) or multivariable analysis (OR = 1.02; 95% CI = 0.50–2.08; *P* = 0.951). We also observed no associations of* CARD8* rs2043211 with individual macrovascular complications (data not shown).

The presence of individual microvascular complications was also investigated, but no association was observed between* CARD8* or* NLRP3* polymorphisms and these complications (Table 2). However, male carriers of at least one polymorphic* NLRP3* rs35829419 allele tended to have more microvascular complications (*P* = 0.050) and had increased risk for end-stage kidney failure (*P* = 0.030, Table 2).

## 4. Discussion

To our knowledge, this is the first study that investigated the influence of genetic variability in inflammasome on T2D long-term complications.

We observed that polymorphic* NLRP3 *rs35829419 A allele is associated with increased risk for development of any macrovascular complications in long-term T2D patients. Despite the small numbers of individuals with a particular complication,* NLRP3 *polymorphism showed statistically nonsignificant association with MI, PAOD, and ICD. These preliminary data are concordant with previous reports on association of* NLRP3* and* CARD8* polymorphism with the proinflammatory phenotype. In a large cohort of healthy blood donors higher IL-1*β* levels were observed in carriers of polymorphic alleles of these genes than in noncarriers [16]. Our results are also in agreement with reports on potential role of NLRP3 inflammasome in MI [21]. Recent genome-wide association study [18] as well as a large cardiovascular candidate gene study [22] observed association of* NLRP3* locus with higher fibrinogen levels that may lead to higher atherothrombotic risk. Inflammasome proteins were indeed shown to be expressed not only in neutrophils and macrophages, but also in vascular endothelia and cardiac fibroblasts [23]. Knockout mice showed declined inflammatory responses and were more protected against myocardial damage after MI [23].

Contrary to our findings, a large First-ever myocardial Infarction study in Ac-county (FIA) showed no association between* NLRP3 *rs35829419 and MI. However, they observed a gender-specific effect of* NLRP3 *rs35829419: polymorphic A allele conferred protection from development of MI in females but was associated with increased CRP levels as a marker of inflammation in males [17]. In our study group, polymorphic* NLRP3* A allele conferred to increased risk of macrovascular complications among males. The risk was highest for PAOD, while a tendency of increased risk of MI was also observed. It was shown that* NLRP3 *may play a role in vascular lipid deposition and atherosclerosis [24].

We observed no associations between any complications and* NLRP3* polymorphisms among females; however, in our study group fewer females than males presented with complications. The discrepancy between our results and the FIA study may be due to the differences in the study design between the two studies: while FIA was a large population based study with cases and controls carefully matched for age and gender [17], our study included patients with T2D as they were coming for their regular follow-up visits and patients with and without complications were not selected for age or gender. As the number of patients with complications was limited, any protective effect of* NLRP3* rs35829419 polymorphism in females may have been missed in our study due to the small numbers. We also have to note that both studies recruited patients from a small geographical area and other factors contributing to MI, such as lifestyle and physical activity, may differ between the two countries. The authors of the FIA study acknowledge that differences in lifestyle factors, including type of food intake and physical activity, between males and females of northern Sweden may contribute to the gender-specific genetic association and MI observed in their study [17].

In our study males also showed some tendency for increased risk of microvascular complications among polymorphic* NLRP3 *rs35829419 A allele carriers, in particular in end-stage kidney failure. Inflammasome was also implicated in inflammatory, autoimmune, and obstructive kidney disease and in ischemia-reperfusion type kidney injury, mostly in rat or mouse models, but the role of NLRP3 inflammasome in diabetic nephropathy remains to be elucidated [25]. Serum IL-18 levels were elevated in patients with diabetic nephropathy, but not in other diabetic complications [26].

We observed no association between* CARD8* rs2043211 and any long-term T2D complications. To our knowledge this is the first study that investigated CARD8 polymorphism in T2D. So far, CARD8 polymorphism was associated with psoriasis [27] and rheumatoid arthritis susceptibility in some [28], but not all, studies [29], but was not associated with juvenile idiopathic arthritis [30].* CARD8* polymorphism did not influence cytokine profiles in a cohort of healthy blood donors [16]. Recently,* CARD8* rs2043211 T allele was found to be significantly associated with a protective effect of ischemic stroke, but not with coronary artery disease [31].

Due to its exploratory nature the present study has several limitations. The study was retrospective in nature and limited to a single regional diabetic outpatient clinic; therefore the number of patients with a particular long-term T2D complication was relatively small. Nevertheless all the patients were clinically well characterized and were followed up by the same diabetologist; therefore, the discrepancies in clinical data collection procedures were minimalized. Furthermore all the patients were recruited in a small geographic area with ethnically homogeneous population; therefore, there was no bias due to genetic heterogeneity [32].

As diabetic complications are multifactorial, we carefully checked for known clinical parameters as possible covariates that could influence our findings. Glycemia was well controlled in all patients, while blood pressure and dyslipidemia were even more tightly controlled in patients with complications to reduce the risk of diabetes-related mortality [33, 34].

*NLRP3 *polymorphism could be used as a molecular marker to identify patients in which pharmacological approach could be used to counteract the proinflammatory phenotype [35]. Furthermore components of NLRP3 inflammasome and downstream signalling pathways are potential novel targets for pharmacological prevention of late complications of T2D [36], as clinical trials targeting other inflammatory cytokines such as IL-6 and TNFa have not been successful [37, 38].

In conclusion, our preliminary data suggest the role of NLRP3 polymorphism in diabetic macrovascular complications, especially in MI. Validation in larger patient cohort is required.

## Figures and Tables

**Table 1:** Patients characteristics.

	All patients (*N* = 181)	Macrovascular complications (*N* = 46)	*P* ^a^	Microvascular complications (*N* = 34)	*P* ^b^
Male gender^c^	105 (58.0)	29 (63.0)	0.423^d^	23 (67.6)	0.206^d^
Age (years)	64.0 (58.5–70.5)	64.0 (58.8–72.3)	0.495	64.0 (58.0–75.3)	0.251
Duration of T2D (years)	11.0 (6.0–17.0)	16.0 (7.0–23.5)	0.003	20.5 (11.0–27.0)	0.000
HbA1c (%) [mmol/mol]	6.9 (6.3–7.6) [52 (45–60)]	6.7 (5.9–7.4) [50 (41–57)]	0.143	6.5 (5.8–7.7) [48 (40–61)]	0.020
BMI (kg/m^2^)	30.0 (28.0–33.3)	30.0 (27.0–33.1)	0.741	29 (25.8–32.4)	0.191
Blood pressure systolic (mmHg)	135.0 (130.0–145.0)	140.0 (128.8–150.5)	0.257	139.0 (119.3–149.0)	0.759
Blood pressure diastolic (mmHg)	80.0 (70.0–80.0)	75.0 (65.8–80.0)	0.014	72.0 (63.0–80.0)	0.000
Total cholesterol (mmol/L)	4.2 (3.5–5.0)	3.9 (3.1–4.5)	0.004	3.7 (2.9–4.7)	0.002
LDL cholesterol (mmol/L)	2.4 (1.9–3.1)	2.3 (1.5–2.7)	0.047	2.0 (1.5–2.4)	0.004
HDL cholesterol (mmol/L)	1.1 (1.0–1.4)	1.1 (0.9–1.3)	0.009	1.1 (0.9–1.4)	0.323
TAG (mmol/L)	1.6 (1.2–2.4)	1.7 (1.1–2.2)	0.587	1.3 (0.9–2.1)	0.046

^a^Comparison between patients with and without macrovascular complications. ^b^Comparison between patients with and without microvascular complications.

Data are shown as median (25%–75%), except for ^c^ *N* (%). *P* values were calculated using Mann-Whitney test, except for ^d^chi square test.

**Table 2:** The association of *NLRP3 *rs35829419 with the risk for macro- and microvascular complications in type 2 diabetes patients.

T2D complications	All patients				Male patients			
	CC *N* (%)	CA + AA *N* (%)	OR (95% CI)	*P*	CC *N* (%)	CA + AA *N* (%)	OR (95% CI)	*P*
Macrovascular	35 (21.9)	11 (52.4)	3.93 (1.54–10.00)	0.004	21 (23.1)	8 (57.1)	4.44 (1.39–14.25)	0.012
PAOD	7 (4.4)	3 (14.3)	3.64 (0.86–15.34)	0.078	4 (4.4)	3 (21.4)	5.93 (1.17–30.06)	0.032
ICD	12 (7.5)	4 (19.1)	2.90 (0.84–10.01)	0.092	9 (9.9)	3 (21.4)	2.48 (0.58–10.60)	0.219
MI	25 (15.6)	7 (33.3)	2.70 (0.99–7.36)	0.052	14 (15.4)	5 (35.7)	3.06 (0.89–10.48)	0.076
Microvascular	28 (17.5)	6 (28.6)	1.89 (0.67–5.29)	0.228	17 (18.7)	6 (42.9)	3.26 (1.00–10.65)	0.050
End-stage kidney failure	20 (12.5)	5 (23.8)	2.19 (0.72–6.63)	0.166	11 (12.1)	5 (35.7)	4.04 (1.14–14.27)	0.030
Retinopathy	12 (7.5)	3 (14.3)	2.06 (0.53–7.98)	0.298	9 (9.9)	1 (7.1)	0.70 (0.08–6.00)	0.746
Neuropathy	12 (7.5)	1 (4.8)	0.61 (0.08–4.97)	0.646	8 (8.8)	3 (21.4)	2.83 (0.65–12.29)	0.165

PAOD: peripheral arterial occlusive disease; 16 (8.85%) ICD: ischemic cerebrovascular disease; MI: myocardial infarction.
